# Diagnostic accuracy of the BD MAX MDR-TB assay on sputum and tongue swabs for *Mycobacterium tuberculosis* complex detection in adults under investigation for TB in South Africa

**DOI:** 10.1128/spectrum.01802-25

**Published:** 2025-11-28

**Authors:** Anura David, Lyndel Singh, Manuel Pedro da Silva, Keneilwe Peloakgosi-Shikwambani, Zanele Nsingwane, Violet Molepo, Wendy Stevens, Lesley Erica Scott

**Affiliations:** 1 Wits Diagnostics Innovation Hub, Health Sciences Research Office, Faculty of Health Sciences, University of the Witwatersrand 37707 , Johannesburg, South Africa; 2 National Priority Programmes, National Health Laboratory Services 70685, Johannesburg, South Africa; London Health Sciences Centre, London, Ontario, Canada

**Keywords:** *Mycobacterium tuberculosis*, BD MAX DR-TB, rifampicin, isoniazid, NAAT

## Abstract

Despite the availability of molecular diagnostics, only 48% of newly diagnosed tuberculosis (TB) cases were confirmed using nucleic acid amplification tests in 2023. The MAX Multi Drug Resistant Tuberculosis (MAX MDR-TB) assay detects *Mycobacterium tuberculosis* complex (MTBC) and resistance to rifampicin (RIF) and isoniazid (INH), but data on clinical performance are limited. This study assessed the assay’s performance on sputum and tongue swabs (TSs). The assay was evaluated for MTBC detection and RIF and INH resistance profiling on sputum using liquid culture as the reference and Xpert MTB/RIF Ultra (Ultra) as a comparator. Diagnostic accuracy for MTBC detection on TS was also assessed. Among 335 participants (56% HIV prevalence), MAX MDR-TB on raw sputum showed an overall sensitivity and specificity of 88.7% (95% confidence interval [CI]: 78.1–95.3) and 98.2% (95% CI: 95.8–99.4) compared with culture and strong agreement with Ultra (Cohen’s kappa = 0.853). A total of 15/55 (27%) sputum samples were classified as “MTB low POS.” Two false RIF-resistant results were observed. INH resistance was missed in two cases. Although specimen numbers were small, TS demonstrated better diagnostic accuracy when using a diluted sample treatment reagent (STR) (66%) buffer. Although the MAX MDR-TB assay demonstrated good agreement with Ultra, Ultra identified more TB cases. Preliminary findings suggest that TS, particularly with a diluted STR buffer, may be a feasible specimen type, but larger studies are required. The high frequency of MTB low POS results highlights the importance of technology assessment by TB programs prior to implementation to minimize repeat testing and enhance diagnostic reliability.

**IMPORTANCE:**

This study evaluates the accuracy of the BD MAXTM MDR-TB assay in detecting *Mycobacterium tuberculosis* complex using sputum and tongue swabs from individuals being assessed for tuberculosis (TB) in South Africa. Rapid and reliable TB diagnosis is crucial for early treatment and preventing transmission. The MAX Multi Drug Resistant Tuberculosis (MAX MDR-TB) assay is a fully automated molecular test that can simultaneously detect TB and drug resistance, offering a potentially faster and more efficient alternative for TB diagnosis. By assessing its performance on different specimen types, this study provides valuable insights into its reliability in clinical settings. Although the MAX MDR-TB assay demonstrated strong agreement with the Xpert MTB/RIF Ultra assay, it identified fewer TB cases. Tongue swabs were also shown to be a feasible specimen type for testing. This research contributes to global TB control efforts by evaluating advanced diagnostics that could streamline testing, reduce delays, and ultimately improve patient outcomes.

## INTRODUCTION

In 2023, tuberculosis (TB) reclaimed its position as the leading infectious cause of death worldwide, causing an estimated 1.25 million deaths (1). Each year, more than 10 million people are newly infected, with diagnosis remaining the weakest link in the TB care cascade (2). Nucleic acid amplification tests (NAATs), such as the Xpert MTB/RIF (Xpert) and Xpert MTB/RIF Ultra (Ultra) (Cepheid, Sunnyvale, CA, USA), have significantly improved TB diagnosis, with the implementation of NAATs showing incremental increases over the years. Despite this progress, in 2023, only 48% of newly diagnosed TB cases were bacteriologically confirmed using a NAAT (1). This underscores the need for broader adoption of molecular diagnostics, many of which are able to detect drug resistance in addition to TB, enabling timely patient management (3).

Apart from molecular WHO-recommended rapid diagnostics and culture, sequencing technologies can be used for drug resistance detection, but their high costs and infrastructure demand currently limit their widespread use. While efforts to reduce these costs and make sequencing more accessible are in progress, the rapidly emerging diagnostic pipeline, with options across the laboratory spectrum (from point of care to highly centralized testing) (4), provides opportunities for more countries to adopt molecular testing.

The WHO-recommended moderate complexity assays (3) enable upfront resistance testing for isoniazid (INH) alongside rifampicin (RIF), facilitating informed treatment decisions at the time of diagnosis. These assays were initially evaluated by our group using spiked sputum (5), and these results, in conjunction with the WHO recommendation, led to diversification of the diagnostic landscape in South Africa (SA)’s TB program (6). However, there are limited clinical data available for these moderate complexity assays.

One of these moderate complexity assays, the BD MAX Multi Drug Resistant Tuberculosis (MAX MDR-TB) assay (Becton, Dickinson and Company, Sparks, MD, USA) was incorporated in the South African TB testing algorithm in 2023 (6). This assay is an automated diagnostic test conducted on the BD MAX instrument (Becton, Dickinson and Company) for detecting *Mycobacterium tuberculosis* complex (MTBC) DNA in raw sputum or sputum pellets (sputum that has been homogenized and decontaminated with N-acetyl-L-cysteine sodium hydroxide). In specimens where MTBC DNA is identified, the assay simultaneously detects mutations in the *rpoB* gene associated with RIF resistance, as well as mutations in the *katG* gene and the *inhA* promoter region, both linked to INH resistance. Clinical performance of the MAX MDR-TB assay on respiratory specimens, reported in literature, demonstrates sensitivity ranging from 80% to 91% and specificity from 97% to 100% (7–9), though some studies were limited by small sample sizes. To the authors’ knowledge, there is only one study, published in 2020, which reported an assay sensitivity of 93% in a South African population, with a 32% HIV-positivity rate (10).

In addition to new innovative technologies, efforts to increase access to TB testing have led to the investigation of additional specimen types, with oral swabs showing improved sensitivity in recent years (11). Downstream testing of oral swabs is typically performed by “in-house” polymerase chain reaction (PCR) or WHO-recommended low-complexity assays (Xpert, Ultra, and Truenat MTB (Molbio Diagnostics, Goa, India). Performance data on oral swabs, specifically tongue swabs (TSs) on other WHO-recommended NAATs are not available.

In this clinical performance evaluation, we aimed to assess the diagnostic accuracy of the MAX MDR-TB assay on sputum for MTBC, RIF, and INH detection compared to a liquid culture reference in a population with a high HIV-positivity rate. Additionally, we assessed the diagnostic accuracy of the MAX MDR-TB assay for MTBC detection in TS specimens (off-label use).

## MATERIALS AND METHODS

### Study design

This cross-sectional, prospective, clinical diagnostic accuracy study evaluated the performance of the MAX MDR-TB assay for MTBC, RIF, and INH detection on sputum. Results were compared to a Mycobacterial Growth Indicator Tube (MGIT) (Becton, Dickinson and Company) liquid culture reference and to Ultra as a comparator. In addition, concordance of the MAX MDR-TB assay on TS was assessed using Ultra, liquid culture, and MAX MDR-TB sputum results as comparators.

### Participant recruitment and study procedures

**Fig 1:**
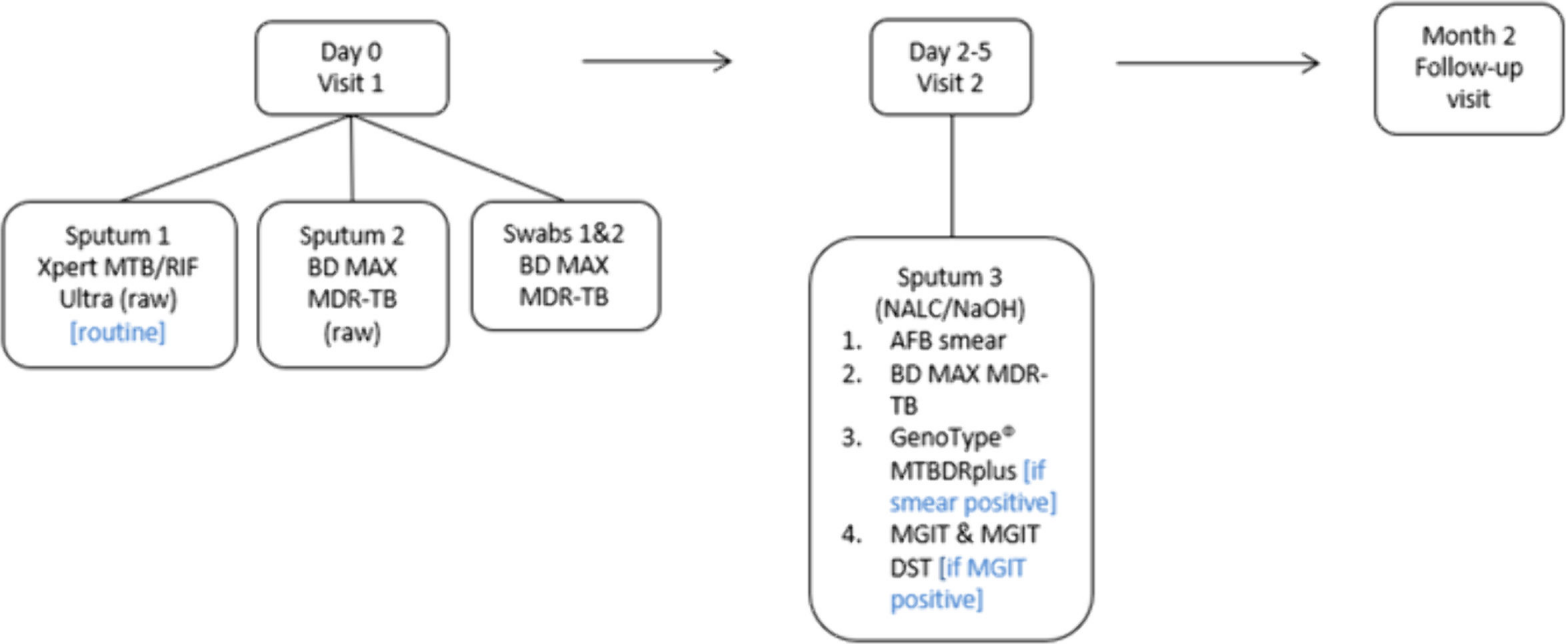
Description of study outline indicating clinic visits and specimen laboratory pathways. AFB, acid-fast bacilli; BD, Becton Dickinson; DST, drug susceptibility testing; MGIT, Mycobacterial Growth Indicator Tube; NALC/NaOH, N-acetyl-L-cysteine–sodium hydroxide.

**Fig 2:**
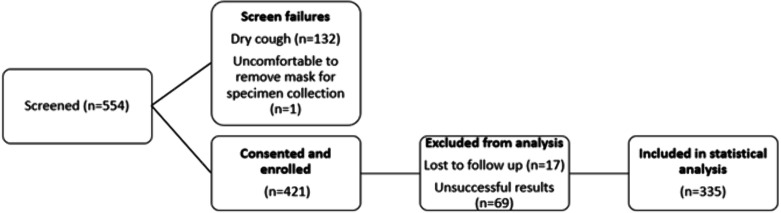
Data description for statistical analysis.

Symptomatic adults (≥18 years), being assessed for TB, were enrolled from 20 October 2021 to 11 July 2023 at the Hillbrow Community Health Centre (HCHC), Johannesburg, SA. The WHO-recommended four-symptom screen—cough, fever, weight loss, and night sweats—was administered to participants. Anthropometric characteristics, including HIV status, TB history, and demographic information, were also collected. Participants’ body mass index (BMI) was also calculated to determine malnutrition, with a BMI of <18.5 indicating undernourishment. To be eligible, participants had to agree to a follow-up visit, provide the required specimens, and have no TB treatment in the 6 months prior to enrollment. Specimen collection occurred over two visits (Fig. 1); at visit 1, two sputum specimens were collected at least 30 minutes apart, with participants refraining from oral intake for at least 30 minutes prior. Two TSs were collected using the procedure described by Andama et al. (12). Swab collection from each participant was performed by research nurses by swabbing the dorsum of the tongue with a Copan FLOQSwab (Copan, Brescia, Italy) for 30 seconds, as far back as possible without initiating a gag reflex. One TS was collected before sputum collection and a second swab after sputum collection. After collection, both TSs were randomly assigned to tubes containing either 1 mL Tris-EDTA (TE) buffer (10 mM Tris-HCl with 1 mM EDTA Na₂, pH 8.0) (Merck, Johannesburg, SA) or no buffer for transport and storage. Sputum was randomized for routine or research testing. At visit 2 (after 2-5 days), routine and research testing were conducted on a single sputum (sputum 3) as indicated in Fig. 2.

Specimens collected for routine and research testing were transported to the Wits Diagnostic Innovation Hub research laboratory in Braamfontein (Johannesburg).

### Routine testing

Only smear-positive sputum (analyzed using auramine-O staining) was tested on the Genotype MTBDR*plus* line probe assay (LPA) (Hain Lifescience/Bruker, Nehren, Germany). All acid-fast bacilli-positive cultures were also tested on the LPA to confirm growth of MTBC. Routine testing and result return were performed by laboratory staff as per the National Tuberculosis TB Management Guidelines (13). Staff were blinded to results from the MAX MDR-TB assay.

### MAX MDR-TB assay testing

Raw sputum and sputum pellets were inactivated using a 2:1 ratio of sample treatment reagent (STR) (Becton, Dickinson and Company) to specimen before testing on MAX MDR-TB, as per manufacturer instructions. Upon receipt at the laboratory, specimens were frozen (sputum at −20°C and TS at −80°C) and then batch tested weekly. For MTBC detection, results are categorized as MTB detected, MTB not detected, MTB low POS (MTBC DNA detected but resistance metrics not measurable), indeterminate (due to BD MAX system failure), incomplete (incomplete run), or unresolved (no MTBC DNA detected and no internal control detected, indicative of an inhibitory sample or reagent failure). For resistance testing, the assay reports resistance detected (RIF or INH resistance mutations were detected), not detected (RIF or INH resistance mutations were not detected), or unreportable (UNR) (MTBC DNA detected but INH or RIF resistance metrics not measurable). Research staff performed testing and were blinded to routine TB results. Swabs were initially tested using the same processing protocol as that used for sputum (*n* = 271). However, parallel testing on contrived swabs showed improved sensitivity using a diluted (66%) STR buffer; hence, diluted STR buffer was used to process the remaining swabs (*n* = 56) (Table 1). Phosphate buffer was used to dilute the STR. Unsuccessful tests were not repeated, as this would compromise the efficiency of rapid diagnostics and incur additional costs.

**TABLE 1:** Tongue swab processing protocol on the MAX MDR-TB assay

STR^*a*^ concentration	Swab transport	Volume of STR added to TS^*b*^ (mL)
Neat (*n* = 271)	Dry	3
	With TE buffer	2
66% (*n* = 56)	Dry	3
	With TE buffer	2

^*a*^ STR, sample treatment reagent.

^*b*^ TS, tongue swab.

### Outcomes and statistical analysis

For performance evaluation, raw sputum and sputum pellet results from MAX MDR-TB were compared with liquid culture for MTBC detection and to phenotypic drug susceptibility testing (pDST) (performed using the MGIT960 SIRE kit; Becton, Dickinson and Company) for RIF and INH resistance detection. Data analysis included calculation of sensitivity, specificity, positive predictive value, and negative predictive value for MTBC detection and concordance for resistance detection, with 95% confidence intervals (CIs) calculated using the Wilson score method (first including all participants and then excluding participants with an Ultra “trace” result). MAX MDR-TB results were additionally compared with Xpert Ultra. To assess the diagnostic accuracy of the MAX MDR-TB assay on TS, concordance with Ultra, liquid culture, and MAX MDR-TB sputum is reported. The target sample size was 400 participants, which was derived using the formula described below with population proportion estimated at 50% and 95% CIs set at 5%. Sample size (*n*) = *Z*^2^ × *p*(1 *p*) / *x*^2^, where *Z* = 1.96, *P* = population proportion, and *x* = confidence interval as a proportion. In determining the sample size, allowance was made for participants who do not meet the inclusion criteria. To determine the performance of the MAX MDR-TB assay, only specimens that generated valid results across all tests (MAX MDR-TB, Ultra, and MGIT) were included in the statistical analysis.

### Follow-up visit

Approximately 2 months after enrollment, participants were contacted telephonically for follow-up. The research nurse evaluated their health status through a series of questions. If the participants reported that they still felt unwell, they were requested to return to HCHC for a follow-up sputum to be collected. Routine testing (smear, MGIT, and any other confirmatory testing) was performed.

## RESULTS

### Study population characteristics

Of all participants screened for enrollment over a 21-month period, 133/554 (24%) were found to be ineligible mainly due to a non-productive cough (Fig. 2). An additional 86 participants were either lost to follow-up or produced unsuccessful test results (either on Ultra, MAX MDR-TB sputum, or MGIT). Statistical analysis was therefore conducted on 335 participants for sputum performance evaluation.

The average age of participants was 39 years, and 213/335 (64%) were male (Table 2). A total of 186/332 (56%) were HIV positive, with three participants reporting unknown HIV status. The prevalence of malnutrition was higher among participants with TB (27/62, 44%) compared to those without TB (62/273, 23%). This difference was statistically significant (Fisher’s exact test; odds ratio, 2.63; 95% CI: 1.46–4.63; *P* = 0.0013). Previous TB was reported by 42/335 (13%) participants. Cough was the most reported symptom, experienced by 334/335 (99%) of participants, with fever being the least reported on 225/335 (67%). Per bacteriological classification using culture, 62/335 (19%) were diagnosed with active TB disease. All participants who were smear positive (all scanty positive) but culture negative showed “MTB not detected” results on Ultra, with only one reporting previous TB. The culture contamination rate was 11% (44/404); of these, Ultra and MAX MDR-TB detected one MTBC-positive specimen, and the participant reported an improvement in symptoms after receiving treatment.

**TABLE 2:** Cohort characteristics of participants used in the statistical analysis

Characteristics	All (*n* = 335)
Demographics	
Age (years), mean (range)	39 (18–70)
Male sex, *n* (%)	213 (63.6)
BMI*^a^* (kg/m^2^), *n* (%)	
<18.5 (underweight)	89 (26.6)
18.5–24.9 (healthy weight)	178 (53.1)
25.0–29.9 (overweight)	41 (12.2)
>30 (obese)	27 (8.1)
HIV-related information, *n* (%)	
HIV positive	186 (55.5)
HIV negative	146 (43.6)
Status unknown	3 (0.9)
TB history, *n* (%)	
Previously diagnosed with TB	42 (12.5)
Clinical signs and symptoms of TB at presentation, *n* (%)	
Cough (any duration)	334 (99.7)
Unexplained weight loss	248 (74.0)
Nights sweats	248 (74.0)
Fever	225 (67.4)
Other^*b*^	244 (72.8)
Bacteriological confirmation (sputum 3), *n* (%)	
Smear and culture positive	45 (13.4)
Smear negative and culture positive	17 (5.1)
Smear and culture negative	269 (80.3)
Smear positive and culture negative	4 (1.2)

^*a*^ BMI, body mass index.

^*b*^ Other symptoms include body aches, loss of appetite, hemoptysis, shortness of breath, dizziness, and vomiting.

### Diagnostic evaluation of the MAX MDR-TB assay on sputum for MTBC detection

The performance of MAX MDR-TB on raw sputum and sputum pellets was compared with liquid culture and, additionally, stratified by HIV and smear status, as outlined in Table 3 for 335 specimen results. Out of the 55 positive MTBC results reported by the MAX MDR-TB assay on raw sputum, 15 (27%) were classified as MTB low POS. For the five raw sputum samples in which MTBC was detected using the MAX MDR-TB assay but not by culture, MTBC was detected by other NAATs in two participants, one by LPA and the other by Ultra. None of the five participants reported previous TB. For the four sputum pellets where MTBC was detected on the MAX MDR-TB assay but not on culture, MTBC was detected on Ultra for one participant. Of these participants, 3/4 (75%) reported previous TB. One of these participants with a positive MAX MDR-TB but negative culture result could not be contacted at the 2-month follow-up visit, while the other eight reported an improvement in symptoms without TB treatment. Unsuccessful results (either indeterminate or UNR) were reported on the raw sputum and/or sputum pellet on 25/403 (6%) participants. The MAX MDR-TB assay yielded a “UNR” result on both specimen types (raw sputum and pellet) for 1/25 (4%) participants.

**TABLE 3:** Performance of smear microscopy, Ultra, and MAX MDR-TB assays on sputum compared to the liquid culture for MTBC detection

Variable	Smear microscopy	Ultra^*e*^ (raw sputum)	MAX MDR-TB (raw sputum)	MAX MDR-TB (sputum pellet)
All (including trace) (*n* = 335)				
Sensitivity (%) (95% CI^*a*^)	72.6 (59.8–83.1)	95.2 (86.5–99.9)	88.7 (78.1–95.3)	87.1 (76.1–94.3)
Specificity (%) (95% CI)	98.5 (96.3–99.6)	97.8 (95.3–99.2)	98.2 (95.8–99.4)	98.5 (96.3–99.6)
PPV^*d*^ (%) (95% CI)	88.2 (76.1–95.6)	90.8 (81.0–96.5)	91.7 (81.6–97.2)	93.1 (83.3–98.1)
NPV^*c*^ (%) (95% CI)	93.0 (89.2–95.7)	98.9 (96.8–99.8)	97.5 (94.8–99.0)	97.1 (94.4–98.7)
All (excluding trace) (*n* = 329)				
Sensitivity (%) (95% CI)	73.3 (60.3–83.9)	95.0 (86.1–99.0)	91.7 (81.6–97.2)	88.3 (77.4–95.2)
Specificity (%) (95% CI)	98.5 (96.2–99.6)	99.3 (97.3–99.9)	98.1 (95.7–99.4)	98.9 (96.8–99.8)
PPV (%) (95% CI)	91.8 (80.4–97.7)	96.6 (88.3–99.6)	91.7 (81.6–97.2)	94.6 (85.1–98.9)
NPV (%) (95% CI)	94.1 (90.7–96.5)	98.9 (96.8–99.8)	98.1 (95.7–99.4)	97.4 (94.8–99.0)
Specimens from HIV-positive individuals (*n* = 186)				
Sensitivity (%) (95% CI)	62.5 (43.7–78.9)	93.8 (79.2–99.2)	78.1 (60.0–90.7)	78.1 (60.0–90.7)
Specificity (%) (95% CI)	98.7 (95.4–99.8)	97.4 (93.5–99.3)	98.7 (95.4–99.8)	98.1 (94.4–99.6)
PPV (%) (95% CI)	91.7 (80.0–97.7)	88.2 (72.5–96.7)	92.6 (75.7–99.7)	89.3 (71.8–97.7)
NPV (%) (95% CI)	94.3 (90.9–96.7)	98.7 (95.3–99.8)	95.6 (91.1–98.2)	95.6 (91.1–98.2)
Specimens from HIV-negative individuals (*n* = 146)				
Sensitivity (%) (95% CI)	85.7 (67.3–96.0)	96.4 (81.7–99.9)	100 (87.7–100.0)	96.4 (81.7–99.9)
Specificity (%) (95% CI)	98.3 (94.0–99.8)	98.3 (94.0–99.8)	97.5 (92.7–99.5)	99.2 (95.4–100.0)
PPV (%) (95% CI)	92.3 (74.9–99.1)	93.1 (77.2–99.2)	90.3 (74.2–98.0)	96.4 (81.7–99.9)
NPV (%) (95% CI)	96.7 (91.7–99.1)	99.1 (95.3–100.0)	100 (96.8–100.0)	99.2 (95.4–100.0)
Smear microscopy negative specimens (*n* = 286)				
Sensitivity (%) (95% CI)	n/a^*b*^	88.2 (63.6–98.5)	70.6 (44.0–89.7)	64.7 (38.3–85.8)
Specificity (%) (95% CI)		97.8 (95.2–99.2)	98.5 (96.2–99.6)	98.5 (96.2–99.6)
PPV (%) (95% CI)		71.4 (47.8–88.7)	75.0 (47.65–92.7)	73.3 (44.9–92.2)
NPV (%) (95% CI)		99.2 (97.3–99.9)	98.1 (95.7–99.4)	97.8 (95.2–99.2)

^*a*^ CI, confidence interval.

^*b*^ n/a, not applicable.

^*c*^ NPV, negative predictive value.

^*d*^ PPV, positive predictive value.

^*e*^ Ultra refers to the Xpert MTB/RIF Ultra assay.

### Comparison of the MAX MDR-TB assay to the Ultra assay for MTBC detection

The MAX MDR-TB and Ultra assays demonstrated a high level of concordance, with an overall agreement of 95.5% (320/335) and a Cohen’s kappa coefficient of 0.853 (95% CI: 0.780–0.925).

Among the discordant results, 10 specimens were positive by Ultra but negative by MAX MDR-TB; all 10 had trace or “very low” semiquantitative results on Ultra, and 5 of these were culture positive. Conversely, five specimens were positive by MAX MDR-TB but negative by Ultra, with one showing MTBC growth on liquid culture.

On raw sputum, there were 4/404 (<1%) unsuccessful results on Ultra compared to 19/403 (5%) unsuccessful results on MAX MDR-TB.

### Detection of drug resistance

#### RIF resistance profiling sputum on the Ultra, liquid culture, and MAX MDR-TB assays

**Fig 3:**
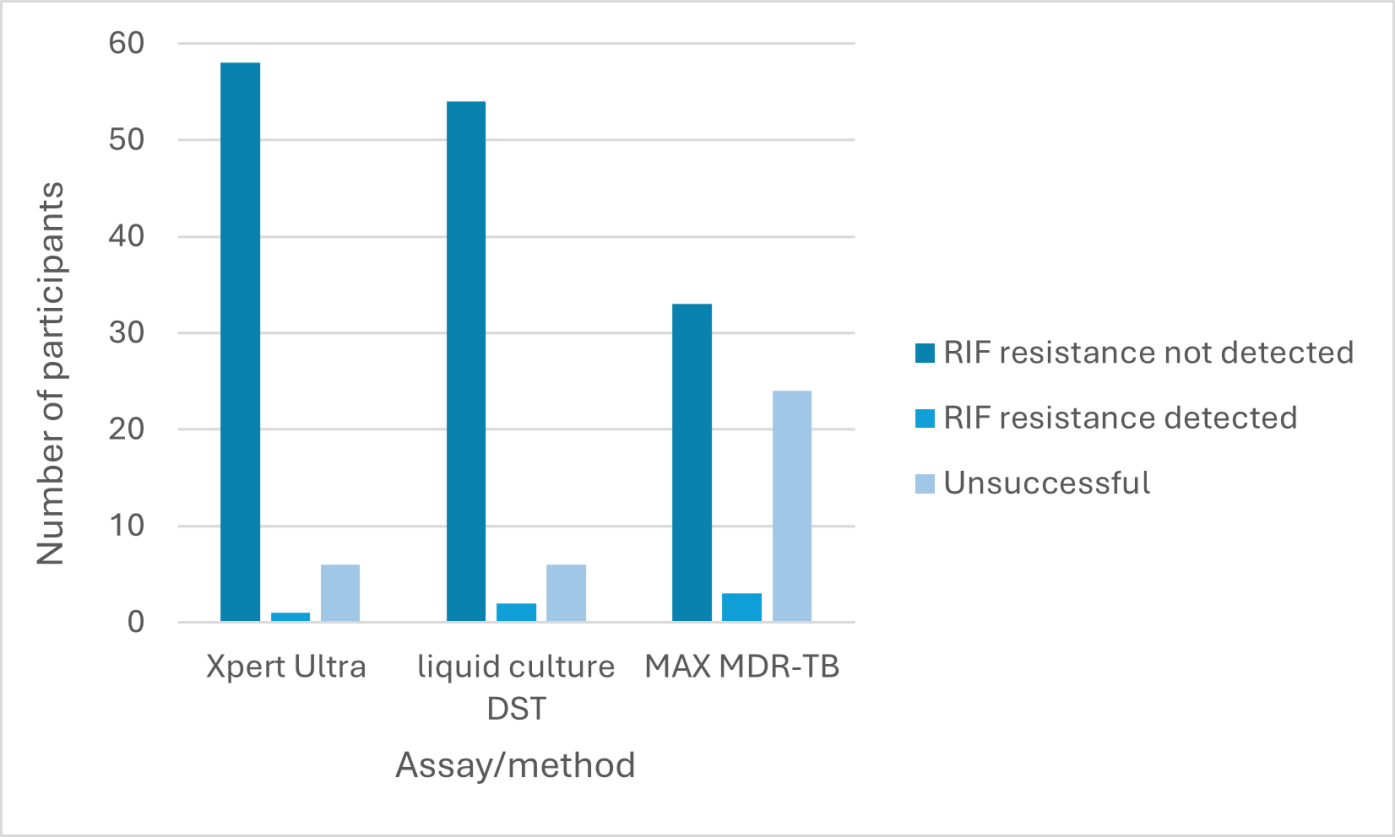
RIF resistance profiles for the Ultra assay, liquid culture, and MAX MDR-TB assay. DST, drug susceptibility testing; RIF, rifampicin. Ultra refers to the Xpert MTB/RIF Ultra assay. MAX MDR-TB refers to the BD MAX MDR-TB assay.

Rifampicin results for Ultra, MAX MDR-TB, and culture are shown in Fig. 3. Unsuccessful RIF results were produced on 6/65 (9%), 6/62 (10%), and 24/60 (40%) sputum for Ultra, culture, and MAX MDR-TB, respectively.

On the MAX MDR-TB assay, valid RIF resistance profiles were available for 35/55 (63%) sputum. When compared to pDST for RIF, the MAX MDR-TB assay, on sputum, correctly assigned resistance profiles to 31/33 (94%) participants (Table 4). Two raw sputum specimens that did not show any resistance on pDST, LPA, MAX MDR-TB sputum pellet, or the Ultra assay were identified as resistant by the MAX MDR-TB assay. Sputum from three participants produced valid RIF results, but INH results were reported as “unreportable.”

**TABLE 4:** MAX MDR-TB rifampicin resistance results compared to phenotypic pDST

pDST^*a*^ result	MAX MDR-TB result			
	RIF-R^*c*^ not detected	RIF-R detected	Indeterminate	Unreportable
RIF^*b*^ sensitive (*n* = 48)	30	2	11	5
RIF resistant (*n* = 1)	0	1	0	0
Unsuccessful (*n* = 6)	2	0	4	0

^*a*^ pDST, phenotypic drug susceptibility testing; Unsuccessful pDST results refer to cultures that failed to grow in the control tube or were repeatedly contaminated.

^*b*^ RIF, rifampicin.

^*c*^ RIF-R, rifampicin resistance.

#### INH resistance profiling for sputum on the MAX MDR-TB assay

Valid INH resistance profiles were available for 38/55 (69%) sputum (Table 5). When compared to pDST for INH, the MAX MDR-TB assay, on sputum, correctly assigned resistance profiles to 34/36 (94%) participants. INH resistance was not detected by MAX MDR-TB on either raw sputum or sputum pellet for two participants, but resistance was detected by pDST. INH resistance was also not detected on the LPA for these participants. Unsuccessful INH results were reported in 17/55 (31%) tests performed. Sputum from five participants produced valid INH results, but RIF results were reported as unreportable.

**TABLE 5:** MAX MDR-TB isoniazid resistance results, compared to phenotypic DST

pDST^*c*^ result	MAX MDR-TB result			
	INH-R^*b*^ not detected	INH-R detected	Indeterminate	Unreportable
INH^*a*^ sensitive (*n* = 45)	33	0	10	2
INH resistant (*n* = 4)	2	1	1	0
Unsuccessful (*n* = 6)	2	0	4	0

^*a*^ INH, isoniazid; Unsuccessful pDST results refer to cultures that failed to grow in the control tube or were repeatedly contaminated.

^*b*^ INH-R, isoniazid resistance.

^*c*^ pDST, phenotypic drug susceptibility testing.

This table compares MAX MDR-TB assay results obtained from TS specimens processed under different conditions (dry or with TE buffer) and compared across specimen processing strategies (neat or 66% diluted STR). Results are expressed as the number of positive results (*n*) within each comparator category (*N*), with percentages in parentheses. Comparator test categories include Xpert MTB/RIF Ultra semiquantitative results (Ultra positive: high, medium, low, very low, and trace; and Ultra negative), liquid culture results (positive or negative for *Mycobacterium tuberculosis* complex/NTM), and MAX MDR-TB sputum results (positive or negative). The table shows concordance across methods and provides a performance overview of TS-based testing relative to multiple comparators.

### Diagnostic accuracy of the MAX MDR-TB assay on tongue swabs

A total of 320 TS were tested on the MAX MDR-TB assay. For those TS processed using neat STR and found to be MTBC positive, 12/19 (63%) and 26/32 (81%) transported “dry” and in TE buffer, respectively, were classified as MTB low POS. When neat STR was used for TS processing, improved detection of MTBC was observed on TS transported in TE buffer compared to those transported dry, regardless of the comparator test (Table 6). When processed with neat STR, TS transported in TE buffer showed MTBC detection in specimens down to a semiquantitative “low” Ultra, whereas TS without buffer only detected MTBC in samples with a semiquantitative result of “medium” or higher. Although neat STR processing demonstrated reduced specificity when TS were transported in TE buffer, a reduced error rate was reported (3% [11/320] compared to 7% [21/320]) for TS that were transported dry. Both sensitivity and specificity seemed improved when using dilute STR, but the sample size was limited on MTBC-positive TS. No MTBC detection was seen on TS from participants who produced a trace sputum result on Ultra (*n* = 6). Of these, two out of six (33%) were confirmed to be bacteriologically positive by culture.

**TABLE 6:** Performance of the MAX MDR-TB assay on tongue swabs compared to Ultra, liquid culture, and MAX MDR-TB sputum results

Comparator test result		MAX MDR-TB TS^*e*^ agreement, *n*/*N* (%)			
		Dry	With TE buffer	Dry	With TE buffer
		Neat STR^*d*^	Neat STR	Dilute STR (66%)	Dilute STR (66%)
Ultra^*f*^ (sputum)	MTBC^*a*^ detected	12/50 (24.0)	20/52 (38.5)	6/9 (66.7)	6/9 (66.7)
	High	10/18 (55.6)	13/19 (68.4)	2/4 (50.0)	2/4 (50.0)
	Medium	2/6 (33.3)	3/6 (50.0)	1/1 (100.0)	1/1 (100.0)
	Low	0/13 (0)	4/14 (28.6)	3/3 (100.0)	3/3 (100.0)
	Very low	0/7 (0)	0/7 (0)	0/1 (0)	0/1 (0)
	Trace	0/6 (0)	0/6 (0)	–^*b*^	–
	MTBC not detected	201/202 (99.5)	204/210 (97.1)	38/38 (100.0)	38/38 (100.0)
Liquid culture	Positive for MTBC	13/47 (27.7)	21/50 (42.0)	6/8 (75.0)	6/8 (75.0)
	Negative for MTBC/NTM^*c*^	205/205 (100.0)	207/212 (97.6)	39/39 (100.0)	39/39 (100.0)
BD MAX MDR-TB (sputum)	Positive for MTBC	13/46 (28.3)	21/48 (43.8)	6/8 (75.0)	6/8 (75.0)
	MTBC not detected	206/206 (100.0)	209/214 (97.7)	39/39 (100.0)	39/39 (100.0)

^*a*^ MTBC, *Mycobacterium tuberculosis* complex.

^*b*^ “–” denotes that there were no specimens in that category.

^*c*^ NTM, non-tuberculosis mycobacteria.

^*d*^ STR, sample treatment reagent.

^*e*^ TS, tongue swab.

^*f*^ Ultra refers to the Xpert MTB/RIF Ultra assay.

When TS results were stratified by collection time relative to sputum collection, independent of TE buffer use or laboratory processing method, MTBC was detected in 24/320 (8%) specimens collected prior to sputum collection and in 27/320 (8%) specimens collected afterward.

## DISCUSSION

This study evaluated the clinical performance of the MAX MDR-TB assay on raw sputum and sputum pellet for MTBC, RIF, and INH detection compared to a liquid culture reference. Ultra was also used as a comparator. Additionally, we assessed the diagnostic accuracy of the MAX MDR-TB assay for MTBC detection on TS specimens.

In this study, ~44% of participants with active TB were undernourished, as indicated by their BMI, which is a TB risk factor (1) and is also associated with increased TB incidence and severity (14). Notably, malnutrition was significantly more common among participants with active TB than among those without, highlighting the strong association between TB and undernutrition in this population.

Assay sensitivity was similar between the sputum pellet (87%) and raw sputum (89%) with MTBC being detected on one additional raw sputum. This falls in the upper end of sensitivity reported in literature (7, 10). Although there was strong agreement (15) between the Ultra and MAX MDR-TB assays, reflecting high overall concordance on raw sputum, Ultra demonstrated better performance, identifying TB in four additional participants. Similar findings were published by Mokaddas et al. (16), where Ultra diagnosed eight additional participants compared to the MAX MDR-TB assay. Raw sputum from almost 30% of participants yielded an MTB low POS result on the MAX MDR-TB assay, which the manufacturer recommends repeating. This translates to an additional cost for the laboratory. For patient management, this means a delayed result, and if the repeat test yields the same result, then this individual needs to return to the healthcare facility to provide a second sputum for resistance testing. Similarly, a trace result on the Ultra assay, observed in six participants in this study population, also requires follow-up testing. These results suggest that nine additional participants may have missed appropriate treatment initiation if the MAX MDR-TB assay was used as the initial TB diagnostic. The MAX MDR-TB assay demonstrated better sensitivity, on raw sputum, in the HIV-negative population (100%) compared to the HIV-positive population (78%). This is not surprising since HIV/TB co-infection is linked to lower bacillary loads, making TB diagnosis more challenging (17).

Of the five potential false-positive results identified by the MAX MDR-TB assay, all participants who could be contacted at the 8 week follow-up reported symptomatic improvement in the absence of any treatment intervention.

The MAX MDR-TB assay offers the advantage of reporting valid resistance results for one drug even if the other is unreportable. However, the assay cannot provide resistance results when an MTB low POS result is generated, as there is insufficient DNA for resistance determination. In this study, an MTB low POS result was reported for a high proportion (27%) of participants, which means that resistance profiles were not available for these participants. For RIF drug-resistance profiling, two false RIF-resistant results were reported by MAX MDR-TB on raw sputum. Some factors contributing to false-positive results can include the detection of non-pathogenic mutations in the *rpoB* gene (18), technical issues within the assay, or mixed infections. Both the MAX MDR-TB assay and the LPA missed INH resistance on two participants. It is possible for phenotypic resistance to be observed before the corresponding genetic mutations are detected. This discrepancy can occur due to limitations in the sensitivity of genotypic assays which typically target the most commonly seen mutations or the presence of resistance mechanisms that are not yet genetically characterized (19). A further consideration is that only a small number of participants with drug-resistant TB were included, which limits the ability to draw definitive conclusions about the assay’s resistance detection performance. The MAX MDR-TB assay was able to produce valid resistance profiles for two participants whose pDST tests were unsuccessful.

For the MAX MDR-TB performance on TS, diagnostic accuracy was chosen over analytical sensitivity and specificity due to the lack of an optimized TS processing method. When using neat STR, TS transportation in TE buffer appeared to improve the sensitivity of the MAX MDR-TB assay with a reduced specificity compared to those that were transported dry. For TS specimens processed with dilute STR buffer, the limited number of MTBC-positive cases precludes drawing definitive conclusions regarding the assay’s ability to detect MTBC. Nonetheless, assay specificity on TS appeared to be higher with dilute STR compared to neat STR, regardless of buffer or dry transportation, with some variability. As with sputum, a notable limitation observed with TS tested on the MAX MDR-TB assay is the high frequency of MTB low POS results. Despite this disadvantage, TS paired with a NAAT could increase access to TB testing, especially in populations who are unable to produce sputum. In this study, 132 participants were ineligible for this study due to a non-productive cough. This population needs to be investigated in future TS evaluation studies as they are likely representative of individuals that are being targeted by initiatives such as Targeted Universal TB Testing (20). An analysis of TS performance, stratified by whether the swab was collected before or after sputum collection, indicates that the timing of TS collection does not impact diagnostic performance. Overall, these findings suggest that TS is a viable specimen type for MTBC detection using the MAX MDR-TB assay. Consistent with observations from WHO-recommended low-complexity assays (21, 22) designed for sputum, MTBC detection from TS is improved in individuals with higher bacterial loads, a trend also observed with the MAX MDR-TB assay.

Ultra produced markedly fewer unsuccessful results (<1%) than MAX MDR-TB (5%) on raw sputum. The high proportion of MTB low POS results, together with observed failures in RIF resistance profiling (40%), underscores the importance of careful technology assessment by TB programs prior to implementation to reduce repeat testing and support reliable diagnosis. These findings highlight that, while the MAX MDR-TB assay shows promise, its MTBC detection rate may be lower than that of other NAATs such as Ultra. This reinforces the need for further optimization and careful evaluation across diverse clinical settings. Future research should focus on improving assay sensitivity and defining its role within integrated TB diagnostic algorithms to ensure accurate and effective patient care.
